# Mechanical behavior of frozen soil improved with sulphoaluminate cement and its microscopic mechanism

**DOI:** 10.1038/s41598-020-73148-3

**Published:** 2020-10-01

**Authors:** Zhenhua Yin, Hu Zhang, Jianming Zhang, Mingtang Chai

**Affiliations:** 1 Northwest Institute of Eco-Environment and Resources, Chinese Academy of Sciences, State Key Laboratory of Frozen Soil Engineering, Lanzhou, 730000 China; 2 University of Chinese Academy of Sciences, Beijing, 100049 China

**Keywords:** Civil engineering, Natural hazards

## Abstract

The foundation of constructions built in the permafrost areas undergo considerable creeping or thawing deformation because of the underlying ice-rich permafrost. Soil improvement may be of advantage in treating ice-rich permafrost at shallow depth. Sulphoaluminate cement was a potential material to improve frozen soil. Simultaneously, two other cements, ordinary Portland cement and Magnesium phosphate cement were selected as the comparison. The mechanical behavior of modified frozen soil was studied with thaw compression tests and unconfined compression strength tests. Meanwhile, the microscopic mechanism was explored by field emission scanning electron microscopy, particle size analysis and X-ray diffractometry. The results showed Sulphoaluminate cement was useful in reducing the thaw compression deformation and in enhancing the strength of the frozen soil. The improvement of the mechanical behavior depended mainly on two aspects: the formation of structural mineral crystals and the agglomeration of soil particles. The two main factors contributed to the improvement of mechanical properties simultaneously. The thicker AFt crystals result in a higher strength and AFt plays an important role in improving the mechanical properties of frozen soils.The study verified that Sulphoaluminate cement was an excellent stabilizer to improve ice-rich frozen soils.

## Introduction

In permafrost areas, an increasing number of engineering constructions have been built, such as roads, railways, buildings and tower footing^1^. They undergo considerable thawing or creep deformation because of the underlying ice-rich permafrost (3–5 m below the permafrost table), which leads to many engineering problems, such as road cracks and building collapse^2–5^. Many cooling measures have been adopted to guarantee the thermal stability of permafrost foundation, such as crushed rock, ventilated ducts, thermosyphons. However, they have a limited cooling extent^6^. With global warming and engineering activities increase, the permafrost foundation will warm or even thaw inevitably. Therefore, it is urgent to seek a more effective way to keep foundation stable even the permafrost thawed, like foundation soil improvement.

The soil improvement is a method to improve the original foundation soil, which makes the foundation soil meet the engineering requirements and greatly reduces the cost, especially in the permafrost areas with limited earthwork resources and high cost of building materials^7–12^. The thawed soil, such as loess and silt, are often treated by chemical stabilizers, including lime, cement, fly ash, enzymes, liquid polymers, resins, acids, silicates, ions, lignin derivatives, etc.^13–15^. In seasonal frozen area, foundation soil improvement is also used to solve the problem of frost heaving^16–18^. In recent years, many researchers have tried to improve permafrost with many kinds of chemical stabilizers. Zhang et al.^19,20^ used the ionic stabilizers to modify frozen soil but the effectiveness was not obvious. Yu et al.^21^ improved the frozen soil with nano-silica, cement and polypropylene fiber and the mixed stabilizer that greatly reduced thaw settlement and increased shear strength. Chai et al.^22,23^ used cement and additives, sodium hydroxide, sodium silicate, sodium lignosulfonate and super-absorbent polymer, etc. to improve frozen soil and the results showed that 15.0% cement and 1.3% Toogood together can lead to a 2.0% thaw compression deformation at 0.1 MPa while the other stabilizers did not achieve the expected effects. Sun et al.^24^ improved frozen soil with Sulphoaluminate cement and decreased the thaw compression strain to 1%. Based on the above studies, it can be concluded that the Sulphoaluminate cement may be an effective stabilizer for the improvement of the shallow foundation in permafrost regions, which can improve significantly the strength of frozen soil after thawing and reduce the thaw compression deformation under the premise of high ice content. However, there are few studies on the application of Sulphoaluminate cement in permafrost, and the types of cement suitable for frozen soil. Meanwhile, the change of mechanical behaviors and the internal mechanism of improved soils are not well understood.

In this paper, frozen soils modified with Sulphoaluminate cement (SAC) and two other cements were tested to investigate the change of strength and deformation behavior. Then, the mechanism controlling the mechanical behavior variations was explained using the methods of field emission scanning electron microscopy (FESEM), particle size analysis (PSA) and X-ray diffractometry (XRD).

## Methods

### Test materials

The test soil was clay collected from Beiluhe Basin on the Qinghai-Tibet Plateau. In laboratory, the soil was air-dried and passed through 2-mm diameter sieve after smashing to ensure the uniformity of specimens. The particle-size distribution, the basic physical and chemical properties of the soil are presented in Table 1. SAC was selected to improve frozen soil. The other two cements, ordinary Portland cement (OPC) and Magnesium phosphate cement (MPC) were selected as the comparison^25^. Both SAC and OPC have been tested before in 42.5 and 52.5. The performances of SAC in the two grades are quite different at low temperature. However, the performances of OPC in the two grades are small differences at low temperature. In order to highlight the types and grades of cement that performs better at low temperature, 52.5 SAC and 42.5 OPC were chosen. The MPC selected in this paper is independently developed by Harbin Institute of Technology. The MPC has no specific cement grade. The main minerals of the SAC and OPC were presented in Table 2. MPC is mainly composed with MgO and K_2_PO_4_ in a certain proportion and the exact composition is unknown.

**Table 1:** Particle-size distribution as well as physical and chemical properties of the soil samples.

Soil type	Particle-size distribution/%				Plastic limit	Liquid limit	pH
Silty clay	> 0.1 mm	0.1–0.05 mm	0.05–0.005 mm	< 0.005 mm	18.6	36.7	8.59
	3.69	11.96	52.83	31.52

**Table 2:** Major components of different cement (%).

Cement type	SiO_2_	Al_2_O_3_	CaO	MgO	SO_3_	Fe_2_O_3_
SAC	12.60	25.80	45.04	1.30	12.60	1.01
OPC	22.12	5.11	61.68	1.06	2.23	5.50

### Sample preparation

Stabilizers were mixed with soil and sifted ice powder at the given moisture content of 33%. The dosages for each kind of cement were respectively set at 5%, 10% and 15% against the mass of wet soil. Meanwhile, 1.0% antifreeze (NaNO_2_) was put into the mixed soil to thaw frozen soil during curing process to start the hydration of cement. In order to promote the hydration of OPC, 0.5% early strength admixture (Na_2_SO_4_) was put in OPC cement-treated frozen soil. Then, the mixed soils were molded in a cylindrical barrel to prepare samples. The sample preparation time should be controlled within 30 min for reducing water loss. Finally, the samples were trimmed and wrapped with several layers of plastic cling film prior to curing for 3, 7, 14 and 28 days in a temperature controlled box at − 1.0 °C. This temperature was chosen in the light of shallow ground temperature in permafrost region in summer. The sample preparation was conducted in − 10 °C environment to prevent the ice from thawing. The percentages mentioned above are percentage of wet soil.

### Thaw compression test

**Figure 1:**
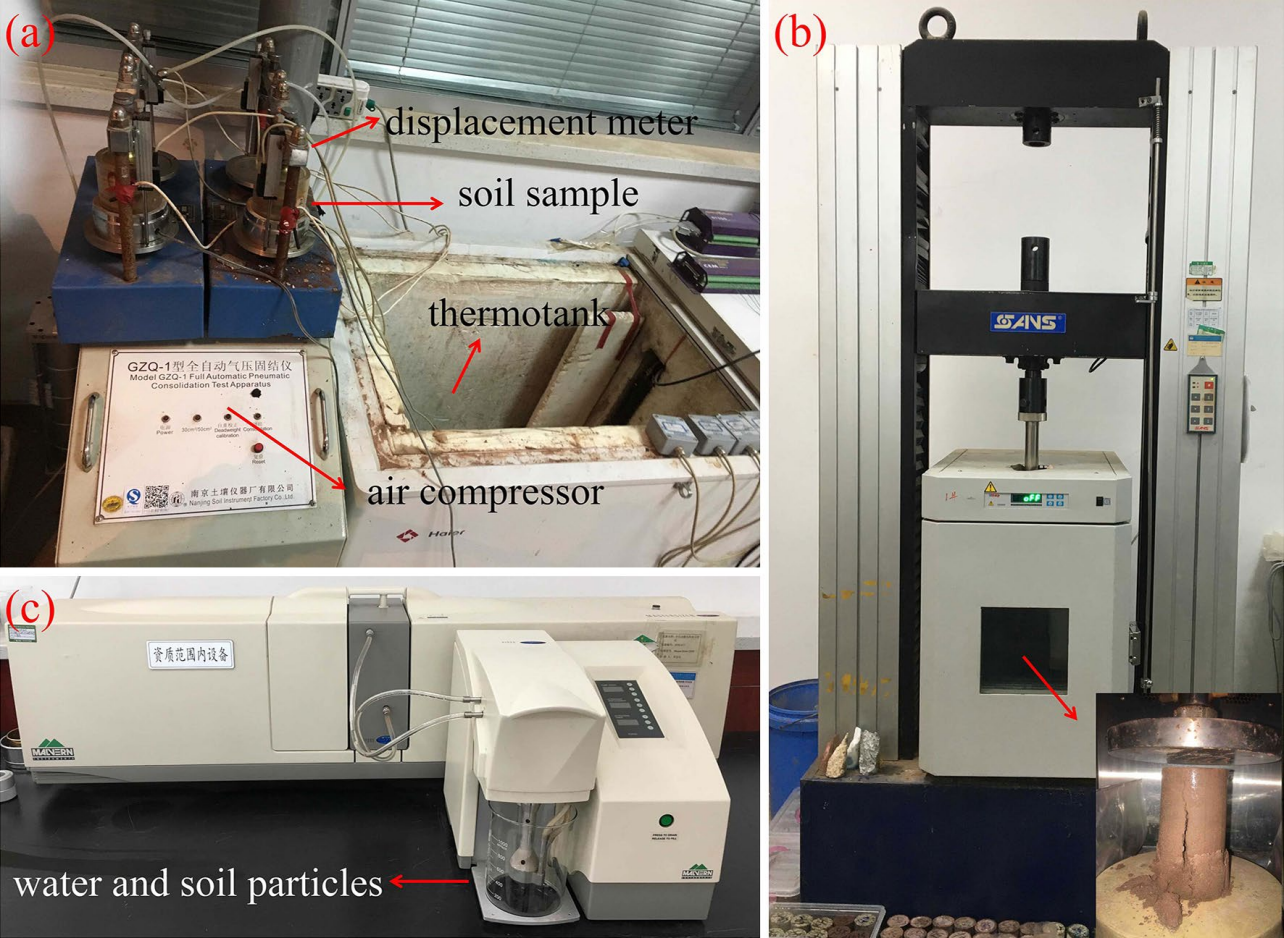
Test apparatuses: (**a)** consolidation apparatus; (**b)** universal testing machine; (**c**) particle size analyzer.

Thaw compression test was conducted to evaluate the effect of the stabilizers on the compressibility of frozen soil. The samples had a size of 61.8 mm in diameter and 126 mm in height. In order to highlight the effect of cement on frozen soil, the dry density was selected around 1.25 g/cm^3^ as initial control condition. It is well known that frozen soil at such a low dry density is extremely compressible. We mixed soil with ice power but water to get a soil sample with such a low dry density. To ensure the reliability of test results, three parallel tests were carried out under each condition. The tests were performed in a consolidation apparatus (Fig. 1a) under a constant load and a step warming temperature condition. The load was 0.1 MPa or 0.2 MPa, and the temperature levels were − 1.0 °C, − 0.5 °C and 2.0 °C. When the deformation rate was less than 0.025 mm/h, the temperature was set to the next step.

### Unconfined compression strength test (UCS)

UCS tests were conducted to access the effect of the stabilizers on frozen soil strength. The samples had a size of 61.8 mm in diameter and 40 mm in height, and the dry density was controlled around 1.25 g/cm^3^. The tests were performed in uniaxial testing machine (Fig. 1b). The axial strain rate was set at 0.01/min and the test temperature was 2.0 °C. An automated data acquisition unit was used to record the applied load and the axial deformation during each test. The strength of each sample was defined by its peak axial stress.

### Field emission scanning electron microscopy test (FESEM)

This method was used to observe the formation of new products in treated frozen soils. After thaw compression test, the specimens were dried in a freeze dryer for 24 h. Small pieces of dried samples were broken off to get fresh sections for observation using FESEM.

### X-ray diffractometry test (XRD)

The samples were scanned in X-ray diffractometer to analyze the types of hydration products and crystalline state of minerals. The dried treated soil was ground and passed a 200-purpose sieve. A small amount of soil was put into the GYS-2 X-ray diffraction analyzer for phase analysis of treated frozen soil. The scanning analysis was adopted the method of continuous scanning measurement. The scanning angle ranged from 10° to 70°. Finally, the collected diffraction result was analyzed.

### Particle size analysis test

Particle size analysis is useful for understanding the modification of soil structure due to chemical reaction. Particle size analyses were performed by using a Mastersizer-2000 particle size analyzer which can measure particles ranging from 0.01 to 10,000 μm, as shown in Fig. 1c. The dried treated soil was ground, passed a 200-purpose sieve, and was put into the beaker of the analyzer. Then, the particle size of the soil was automatically analyzed and collected.

## Results

### Thaw compression deformation

**Figure 2:**
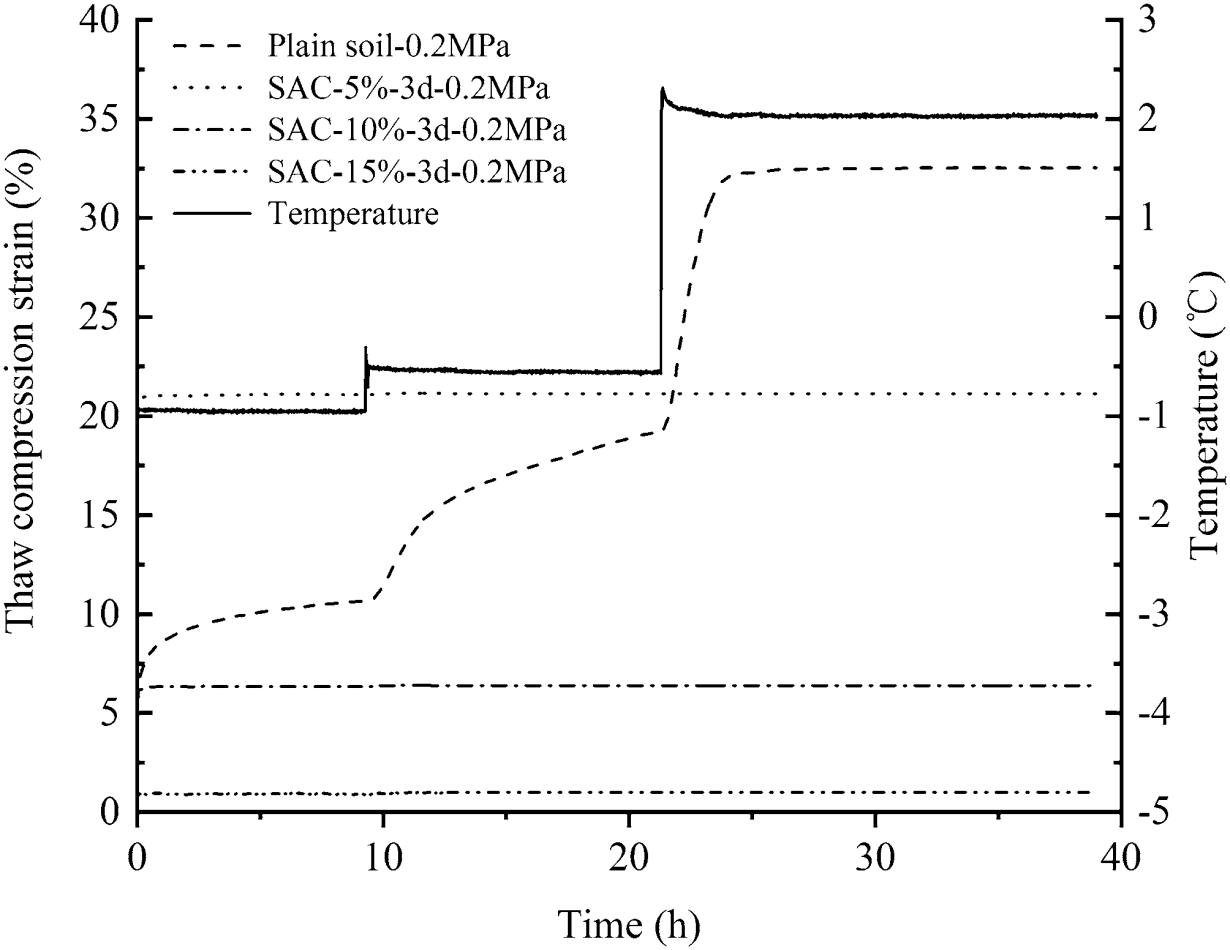
Thaw compression deformation curves of the frozen soil treated with SAC.

Figure 2 gives an example of thaw compression curves of untreated sample and SAC samples cured for 3 days under the load of 0.2 MPa. It is obvious that untreated sample presented a remarkable creep deformation at one temperature level and an abrupt increase in deformation as the raised temperature. The deformation increased rapidly to the maximum of 32.5% when the temperature warmed to 2.0 °C, showing a strong thaw compression deformation behavior as expected. The SAC samples developed immediately a compressive deformation when they were loaded by 0.2 MPa. Then, the deformation curves kept constant with increasing temperature, in that the additive of antifreeze thawed completely the soils even at − 1.0 °C. Compared to the untreated sample, the SAC samples had a remarkable ability against thaw compression deformation. However, the difference in SAC dosage gave a large difference in decrease of thaw compression. The maximum strain of the sample with 5% dosage of SAC dropped to 21.2%, 6.5% for 10% dosage and 1.0% for 15% dosage, which signifies that addition of SAC at an appropriate dosage can effectively reduce or even eliminate the thaw compression deformation of frozen soils.

**Figure 3:**
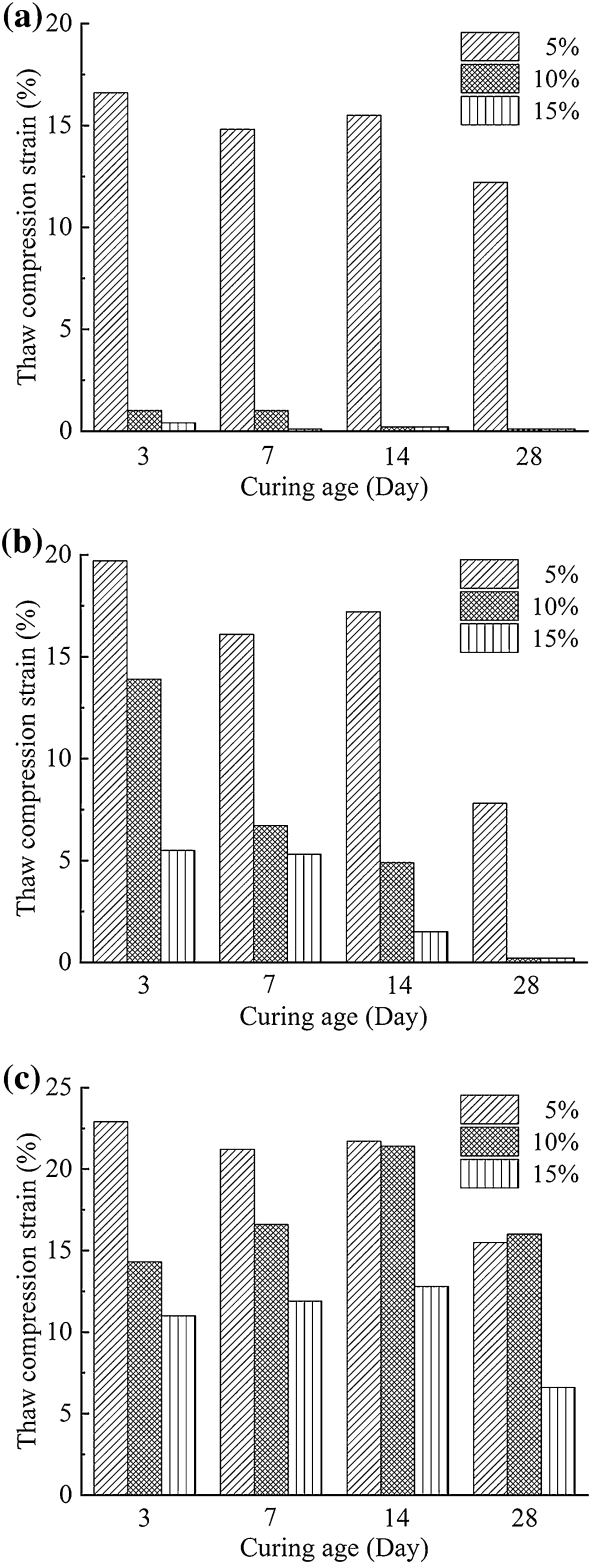
Compression strain of frozen soil treated with SAC, OPC and MPC under 0.1 MPa after different curing ages: (**a**) SAC; (**b**) OPC; (**c**) MPC.

Figure 3 shows the thaw compression results on treated samples at dosages of 5%, 10% and 15% after curing ages of 3, 7, 14 and 28 days, respectively. The samples were all loaded by 0.1 MPa and the final temperature was increased to 2.0 °C. The results generally revealed that the treated soils with 5% stabilizers developed a considerable level of compression deformation, and more dosage and longer curing age caused greater reduction in deformation. For the SAC soils, the strain declined from 16.6% to 0.4% by increasing cement dosage from 5% to 15% when curing time is 3 days, and it dropped from 1.0% to 0.1% by prolonging the curing time from 3 to 28 days when the cement dosage is 10%. The OPC soils could also reach a fairly small strain 0.2% after 28 curing days when it had a cement dosage of 10%. The increase in dosage gave a marked effectiveness in enhancing the capability to resist deformation, for instance, the strain decreased from 16.1% to 5.5% as the dosage increased from 5% to 15% after 14 curing days. Apparently, OPC soils showed good effect in improving the deformation resistance but had a poorer effectiveness than SAC soils. Compared to these two soils, MPC had the poorest ability to improve the frozen soil. The best effect on improving frozen soil occurred at the dosage of 15% and the curing age of 28 days. However, there was still a great compression strain of 6.6%. Consequently, SAC may be a favorable stabilizer in eliminating the compression deformation of frozen soils, even with a smaller dosage (e.g. 10%) and a shorter curing time (e.g. 14 days).

**Figure 4:**
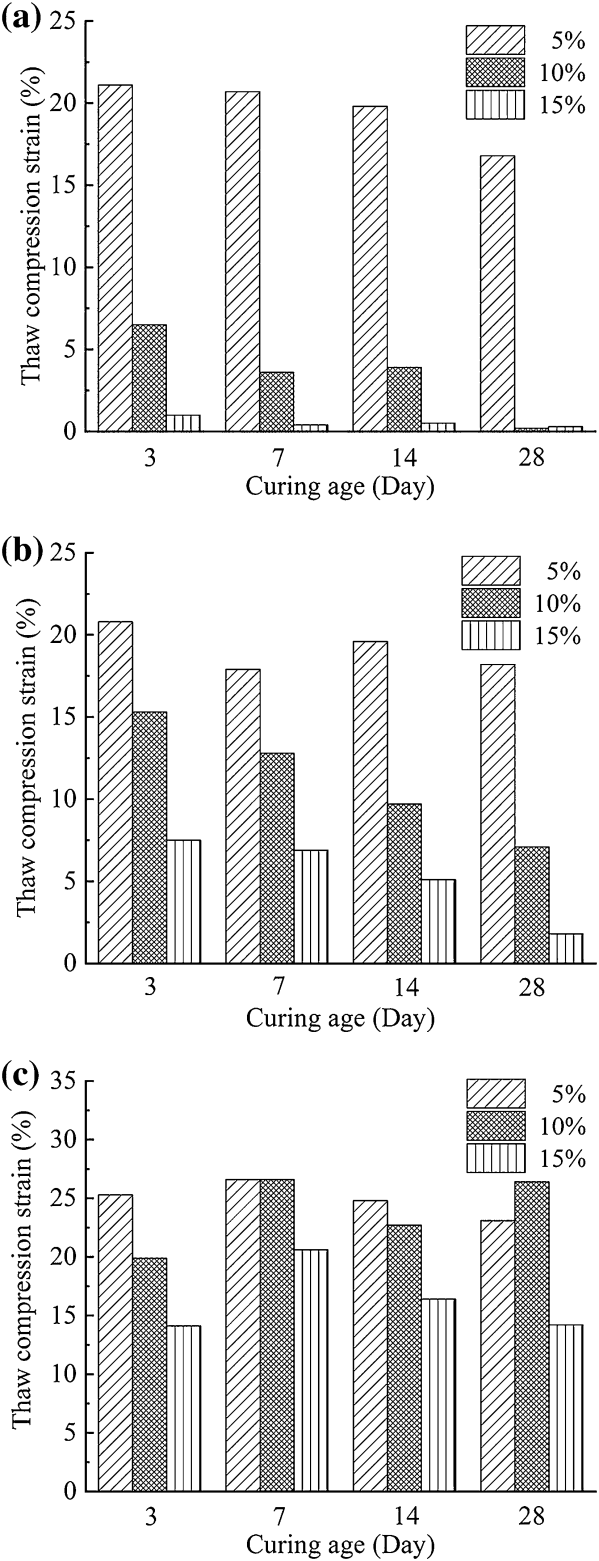
Compression strain of frozen soil treated with SAC, OPC and MPC under 0.2 MPa after different curing ages: (**a**) SAC; (**b**) OPC; (**c**) MPC.

When the samples were loaded by 0.2 MPa, they presented similar variation tendency in compression deformation with the result under 0.1 MPa, with increasing cement dosage and prolonging curing time (Fig. 4). However, the load increment largely increased the compression deformation, except for the SAC soil with more than 10% dosage or after 28 curing days.

### Unconfined compression strength

**Figure 5:**
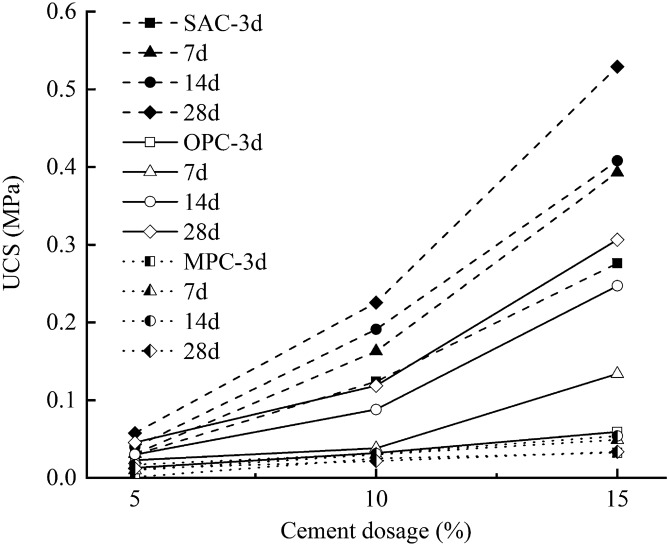
UCS of treated frozen soil with SAC, OPC and MPC under different curing ages.

Figure 5 shows the results of UCS test on SAC, OPC and MPC samples with dosages of 5%, 10% and 15% after curing age of 3, 7, 14 and 28 days, respectively. Apparently, the samples had the UCS much smaller than 0.1 MPa when they were treated with 5% dosage of cements. The strengths of the samples were reinforced with increasing dosage and curing time. However, the addition of the MPC enhanced indistinctively the strength of the soils that were overall far less than 0.1 MPa, even when the cement dosage and curing age increased. The OPC seemed have a better effect on improving the soil strength than the MPC. Their strength increased appreciably in response to the increase of cement dosage and curing time, but basically no more than 0.3 MPa. By comparison, the SAC had the most remarkable effectiveness on enhancing the soil strength. The sample treated with 15% dosage of SAC and cured by 28 days reached an unconfined strength of 0.53 MPa, which could meet the requirement on rolling compaction strength of subgrade backfilling^26^. In practical engineering, the cements will be applied to mix the shallow permafrost to reinforce the subgrade stability once the ground thaws, thus the tests were performed at a positive temperature of 2.0 °C. Compared to the natural permafrost with lots of ice that will lost strength once the ground thaws, the frozen soil treated with SAC had a great ability to resist the overlying load.

### Microstructural characteristic of treated soils

**Figure 6:**
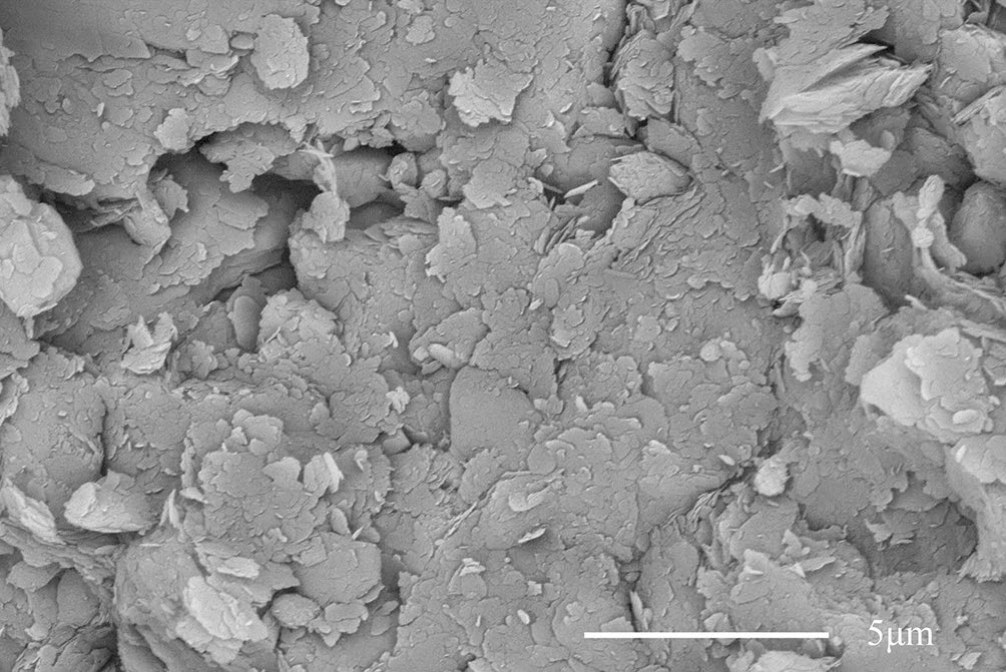
Micrograph image of untreated frozen soil.

Microstructural characteristic can provide powerful evidence for explaining the change in the mechanical behavior of the soils treated with cement. Figure 6 shows the microscopic picture of untreated frozen soil. It showed a scattered, overlapping and irregular lamellar structure, which is the typical characteristic of the clay used in this study^22^.

**Figure 7:**
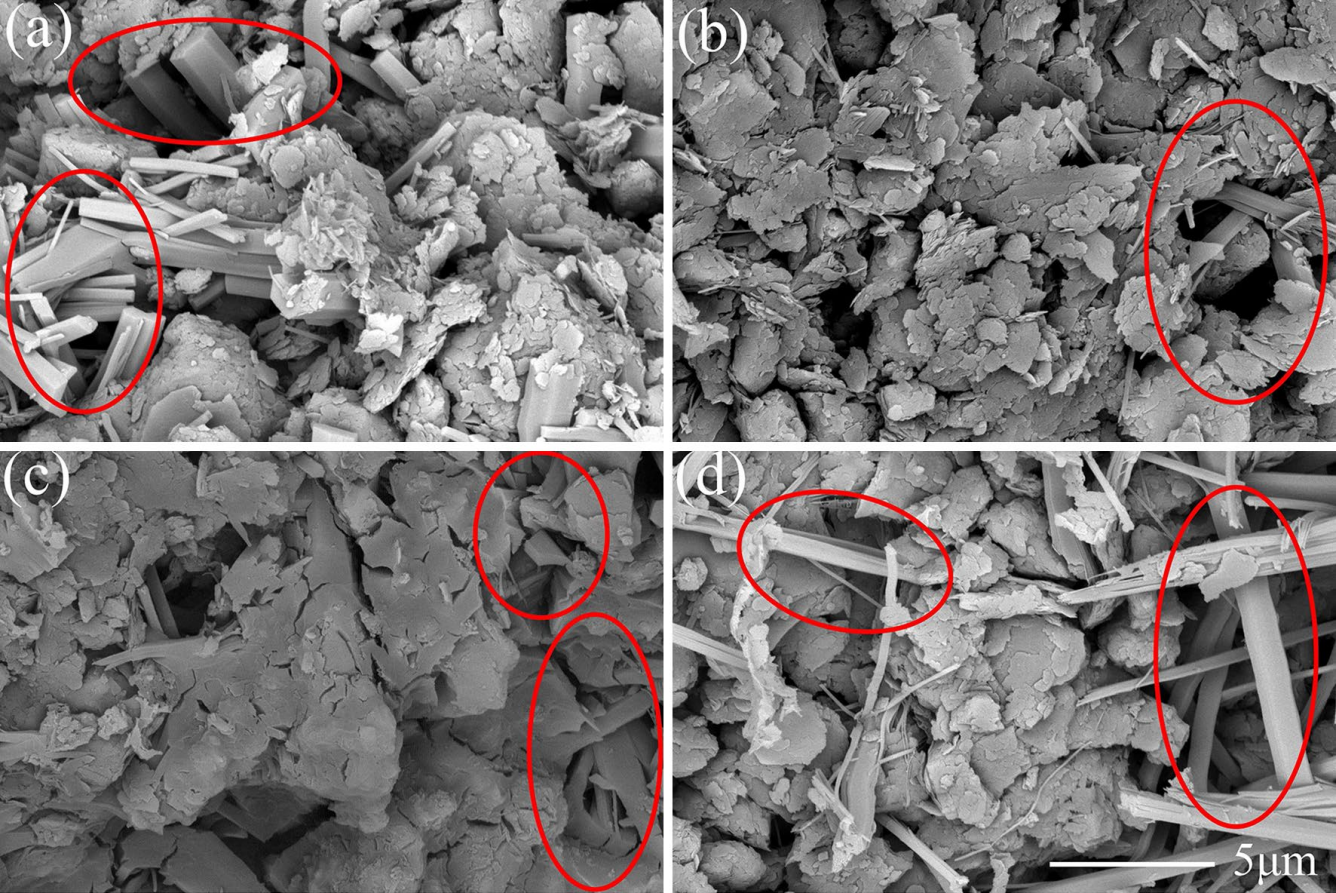
Microscopic images of treated soil with 15% SAC after different curing ages: (**a**) 3 days; (**b**) 7 days; (**c**) 14 days; (**d**) 28 days;

**Figure 8:**
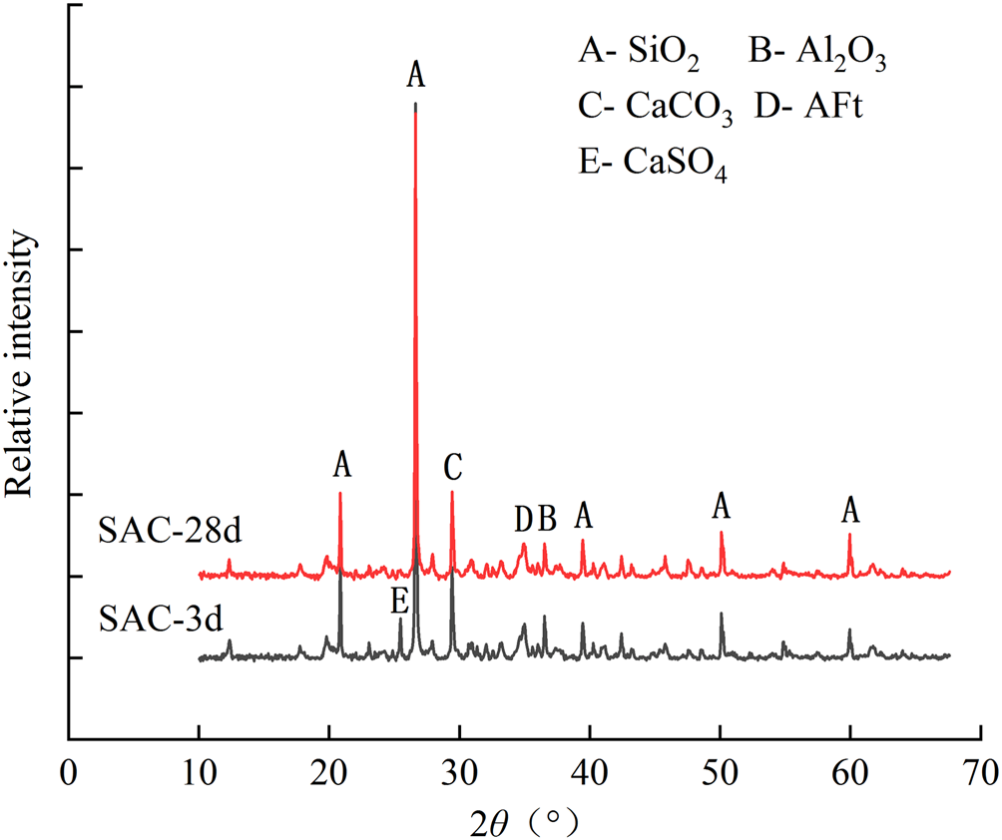
XRD curves of treated soils with 15% SAC.

Figure 7a–d present the microscopic pictures of SAC samples with the dosage of 15% after the curing age of 3, 7, 14 and 28 days, respectively. Figure 8 illustrates the XRD diffractometry curves of SAC samples at the dosage of 15% under the curing time of 3 and 28 days. The main chemical reactions of SAC are as follows.

1 $${\text{C}}_{4} {\text{A}}_{3} {\overline{\text{S}}} + 2{\text{C}}{\overline{\text{S}}}{\text{H}}_{2} + 34{\text{H}} \rightarrow {\text{C}}_{3} {\text{A}} \cdot 3{\text{C}}{{\overline{\text{S}}}} \cdot {\text{H}}_{32} + 2{\text{AH}}_{3}$$

2 $${\text{C}}_{4} {\text{A}}_{3} {\overline{\text{S}}} + 2{\text{C}}{{\overline{\text{S}}}} + 38{\text{H}} \rightarrow {\text{C}}_{3} {\text{A}} \cdot 3{\text{C}}{{\overline{\text{S}}}} \cdot {\text{H}}_{32} + 2{\text{AH}}_{3}$$

3 $${\text{C}}_{2} {\text{S}} + 2{\text{H}} \rightarrow {\text{C}} - {\text{S}} - {\text{H}} + {\text{CH}}$$

According to the reaction mechanism, the main hydration products of SAC are polysulfide calcium sulphoaluminate hydrate (AFt), aluminum hydroxide gel (AH_3_) and calcium silicate hydrate gel (C-S–H). The AFt presents needlelike and pillared morphological characteristics^27^. When the gypsum is exhausted, the aluminate ions react with AFt giving the monosulfide calcium sulfoaluminate which is known as AFm^28^. According to the reference, AFm and C–S–H is almost amorphous, so it cannot be observed in SEM images and XRD results^29^. The XRD diffractometry result showed AFt were formed during curing. Thus, the pillared crystals circled by red line in Fig. 7 could be determined as AFt. At the curing age of 3 days, the microscopic image showed that much AFt had been generated Fig. 7a. Meanwhile, XRD results show a peak value of gypsum (E), indicating that the gypsum has not been used up at this time. At 28 days, the diffraction peak of the gypsum has disappeared, indicating that the gypsum has been exhausted and AFt had partially converted into AFm. However, at the curing age of 28 days, there were still a large number of AFt crystals in the image, and there was also an obvious AFt peak in XRD result. It indicated only a small part of AFt had converted to AFm.

**Figure 9:**
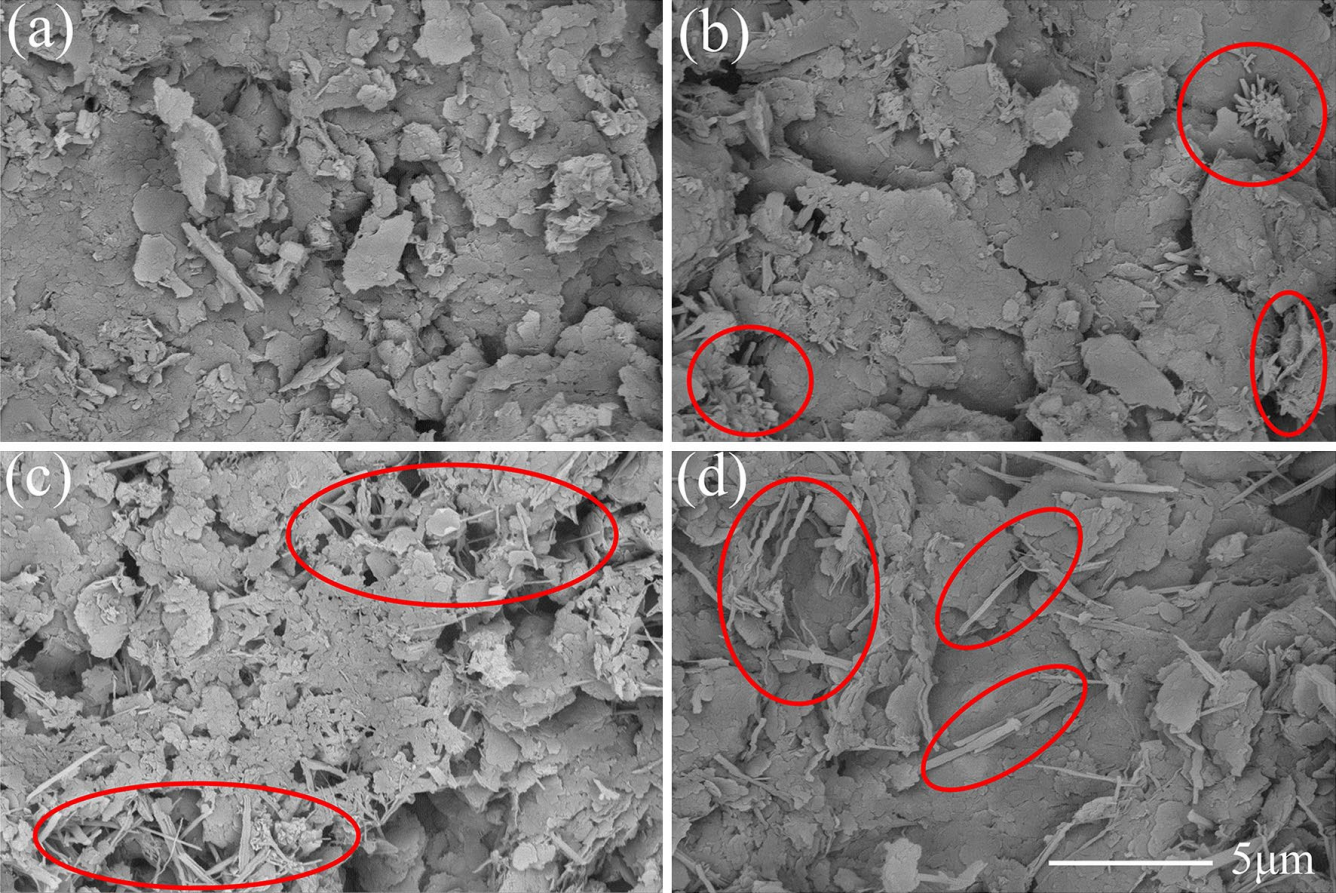
Microscopic images of treated soil with 15% OPC after different curing ages: (**a**) 3 Days; (**b**) 7 Days; (**c**) 14 days; (**d**) 28 days.

**Figure 10:**
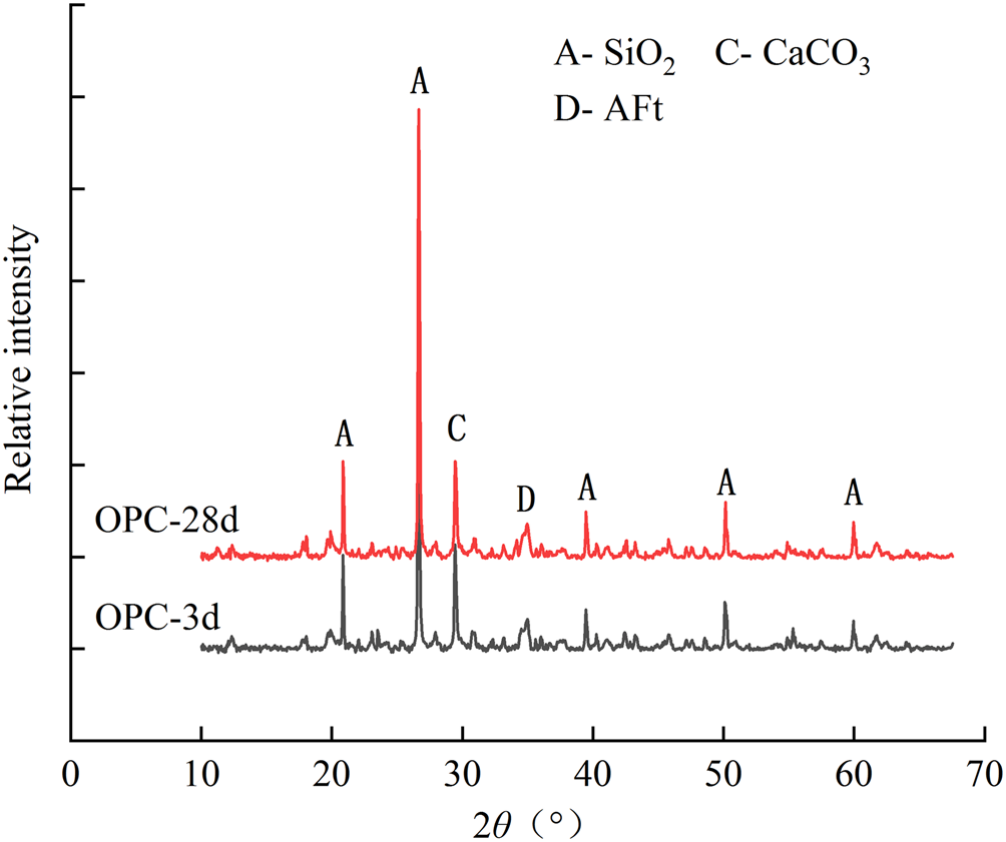
XRD curves of treated soils with 15% OPC.

Figure 9a–d present the microscopic pictures of OPC samples with the dosage of 15% after the curing age of 3, 7, 14 and 28 days, respectively. Figure 10 shows the XRD curves of OPC samples at the dosage of 15% after the curing time of 3 and 28 days. The main chemical reactions of SAC are as follows.

4 $${\text{C}}_{3} {\text{S}} + 6{\text{H}} \rightarrow {\text{C}} - {\text{S}} - {\text{H}} + 2{\text{CH}}$$

5 $${\text{C}}_{2} {\text{S}} + 2{\text{H}} \rightarrow {\text{C}} - {\text{S}} - {\text{H}} + {\text{CH}}$$

6 $$2{\text{C}}_{3} {\text{A}}\overline{\text{S}} + 3{\text{C}}{{\overline{\text{S}}\text{H}}}_{2} + 26{\text{H}} \rightarrow 2{\text{C}}_{3} {\text{A}} \cdot 3{\text{C}}{{\overline{\text{S}}}} \cdot {\text{H}}_{32}$$

The hydration products of OPC are similar to those of SAC. According to the reaction mechanism, the main hydration products of OPC are AFt and C-S–H. The AFt was also found in the microscopic images and XRD result. However, the AFt presented a short needlelike shape. At the curing age of 3 days, the microscopic image in Fig. 9a showed that there were no obvious AFt crystals in the soil sample. With the raised curing ages, AFt crystals gradually increased. At the curing age of 28 days, the image in Fig. 9d showed the characteristics of compactness and integrity. The diffraction peak of AFt also proved the extence of AFt (Fig. 10).

**Figure 11:**
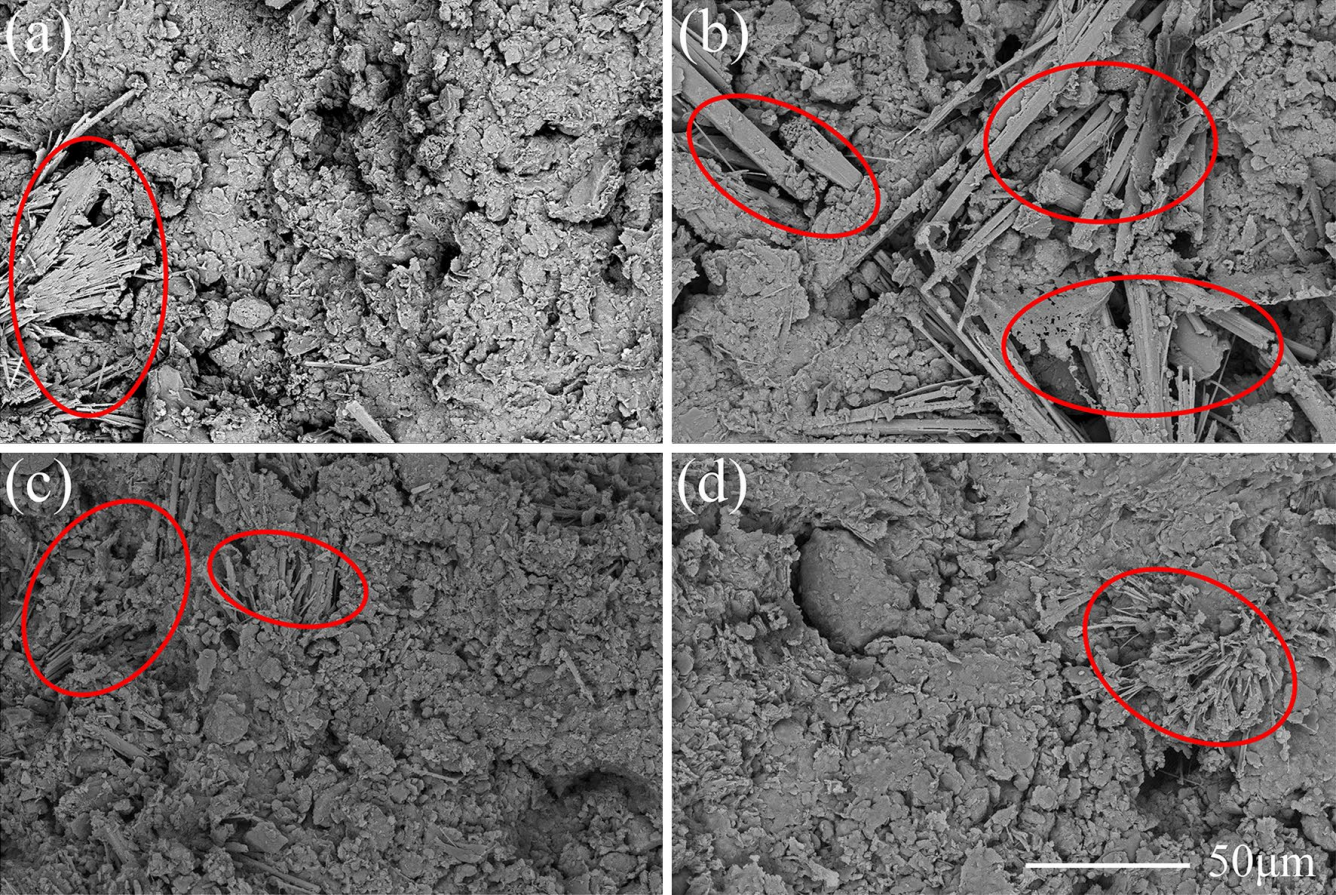
FESEM results of treated frozen soil with 15% MPC under different curing ages: (**a**) 3 days; (**b**) 7 days; (**c**) 14 days; (**d**) 28 days.

**Figure 12:**
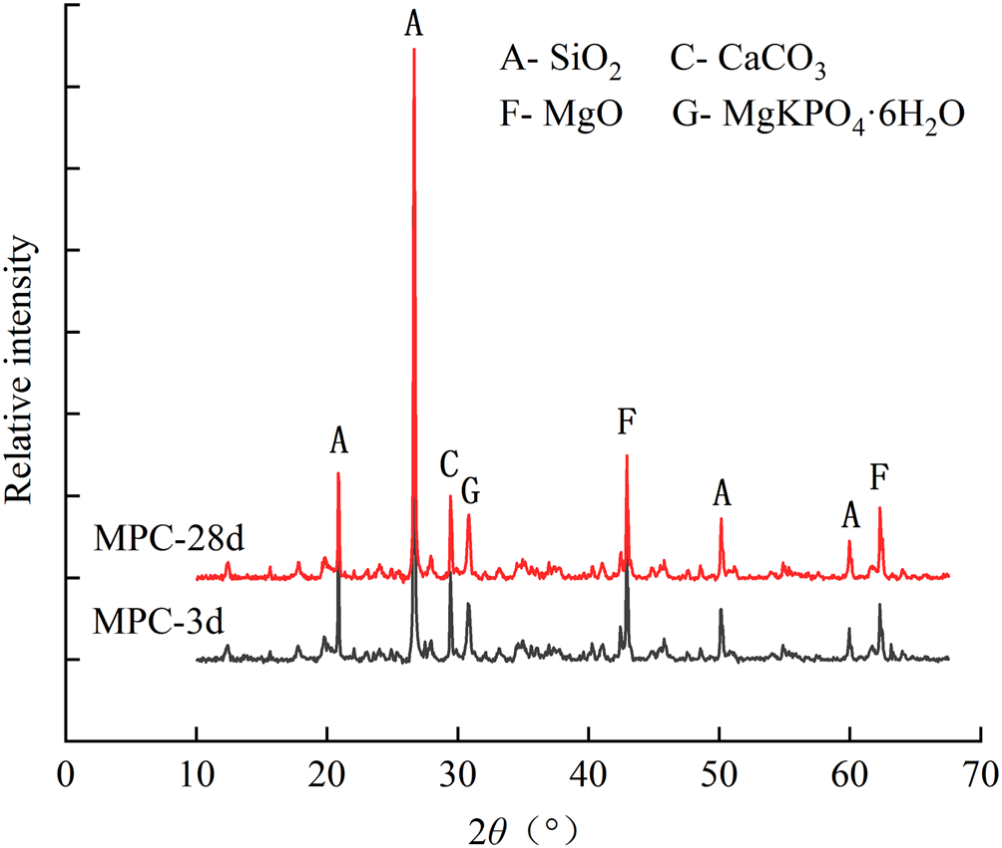
XRD of treated soils with 15% MPC.

Figure 11a–d present the microscopic pictures of MPC samples with the dosage of 15% after the curing age of 3, 7, 14 and 28 days, respectively. Figure 12 shows the XRD curves of MPC samples at the dosage of 15% after the curing time of 3 and 28 days. The reaction mechanism of MPC is that magnesium oxide reacts with phosphate in solution to form hydration gel struvite (MgKPO_4_·6H_2_O). The gel enfolds the excess MgO particles, resulting in a stronger MPC. According to the previous publication and the XRD result, the crystal with the clubbed and sheet shapes is magnesium phosphate potassium hydrate (MKP)^30^. Figure 11a–d all showed the clubbed and sheet MKP crystals. However, the distribution of MKP crystals in the soil is random. The MKP crystals cured for 7 days look bigger than others. Actually, large and small crystals both appeared in the soil sample in the different location. According to the hydration reaction mechanism, the MKP crystals do not change further. The diffraction peaks of MKP were almost no difference at different curing ages (Fig. 12).

Comparing the SEM images Figs. 7, 9 and 11, it seemed that the more porous for the microstructure of the SAC samples than that of the OPC samples and MPC samples. It was because that the cement improved samples in the SEM tests were the samples after thaw compression at different curing times and consolidation pressure. The initial porosity was similar. At the same cuing time and the same consolidation pressure, SAC sample had little or no compression deformation, and the porosity was large. However, the thaw compression deformation of OPC sample was large and the porosity was small. MPC sample had the largest thaw compression deformation and the smallest porosity.

### Particle size analysis

**Figure. 13:**
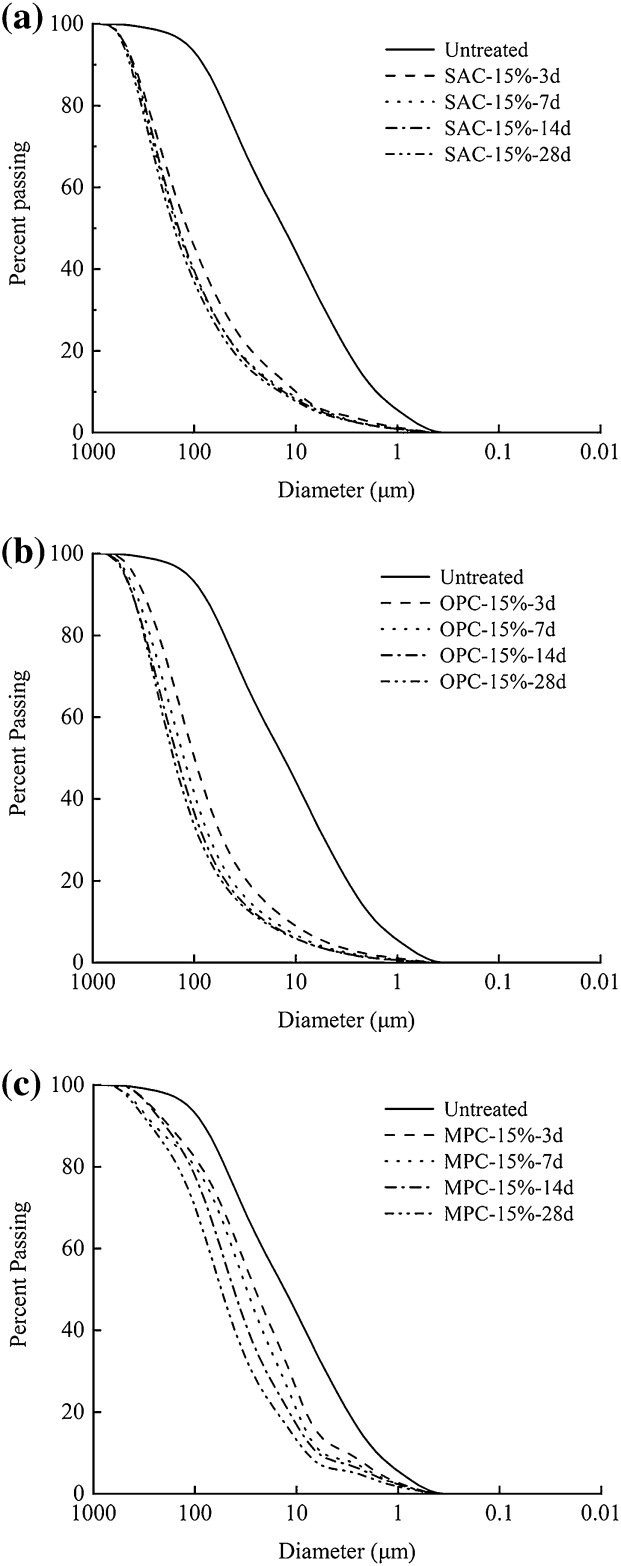
Particle size analysis of untreated and treated soils with 15% SAC, OPC and MPC under different curing ages (**a**) SAC; (**b**) OPC; (**c**) MPC.

Figure 13 shows the particle size analysis curves of untreated and treated soils. Apparently, the particle sizes of the treated soils were much larger than that of the untreated soil, and became greater with increasing curing time. However, the extent of the particle sizes change depended on the kind of the cement. The untreated soil had a particle size distribution that the particles with the diameter smaller than 0.002 mm accounted for 12% and those between 0.002 mm and 0.075 mm accounted for 88%. For the soils treated with 15% SAC for 28 days, the amount of the particles with diameter less than 0.002 mm decreased to 2% while those between 0.002 mm and 0.075 mm decreased to 30%. The OPC samples exhibited a similar change in particle size distribution to SAC samples. When treated with 15% OPC for 28 days, the percentage of the particles smaller than 0.002 mm dropped to 2% while those between 0.002 mm and 0.075 mm dropped to 26%. These two kinds of cement agglomerated considerably the particles through hydration reaction. MPC had the lowest ability in agglomerating soil particles, in that the two percentages changed to 4% and 60% when the soil treated with 15% OPC for 28 days.

## Discussion

High ice content is the critical cause of a sharp thaw settlement when the permafrost subgrade is disturbed to be thawed. Getting rid of the ice-rich permafrost, like replacement of coarse granular soils, is a well-advised method in ensuring the long-term stability of the engineering structures. However, the replaced materials are hard to get in cold regions. Thus, the treatment of the ice-rich permafrost with stabilizers becomes an optimal way to ensure the soil strength meeting engineering requirement. The water or ice in permafrost reacts with stabilizers so as to improve the soil strength even the geotemperature warms to positive.

In our study, the samples treated with SAC and OPC showed great mechanical properties when they were cured for 28 days. The macroscopic behavior is related closely to the development of microstructure. For the two kinds of treated samples, some similar microscopic characteristics were found in microscopic images and particle size analysis results. The two cements improved soil were manifested the generation of a large number of AFt crystals through hydration reaction. The pillared or needlelike AFt constituted a skeleton structure in soil, which reinforced effectively the soil. Meanwhile, the cementitious hydration products agglomerated remarkably the soil particles, causing a sharp decrease of the particles between 0.002 mm and 0.075 mm from 88% to less than 30% after 28 curing days. The two main factors contributed to the improvement of mechanical properties simultaneously. By contrast, although a large of MKP crystals had generated in the MPC improved frozen soil, the gel products did not agglomerate the soil particles well and the soil particles only increase to a small extent. Therefore, the strength of frozen soil had hardly improved. This conclusion is similar to the previous study that the soil solidified with phosphate cement had a low strength at negative temperatures^25^. Through the comparison of three kinds of cement in this paper, it can be known that the strength enhancement of cement improved soil is mainly based on the crystal mineral and cementitious products simultaneously.

**Figure 14:**
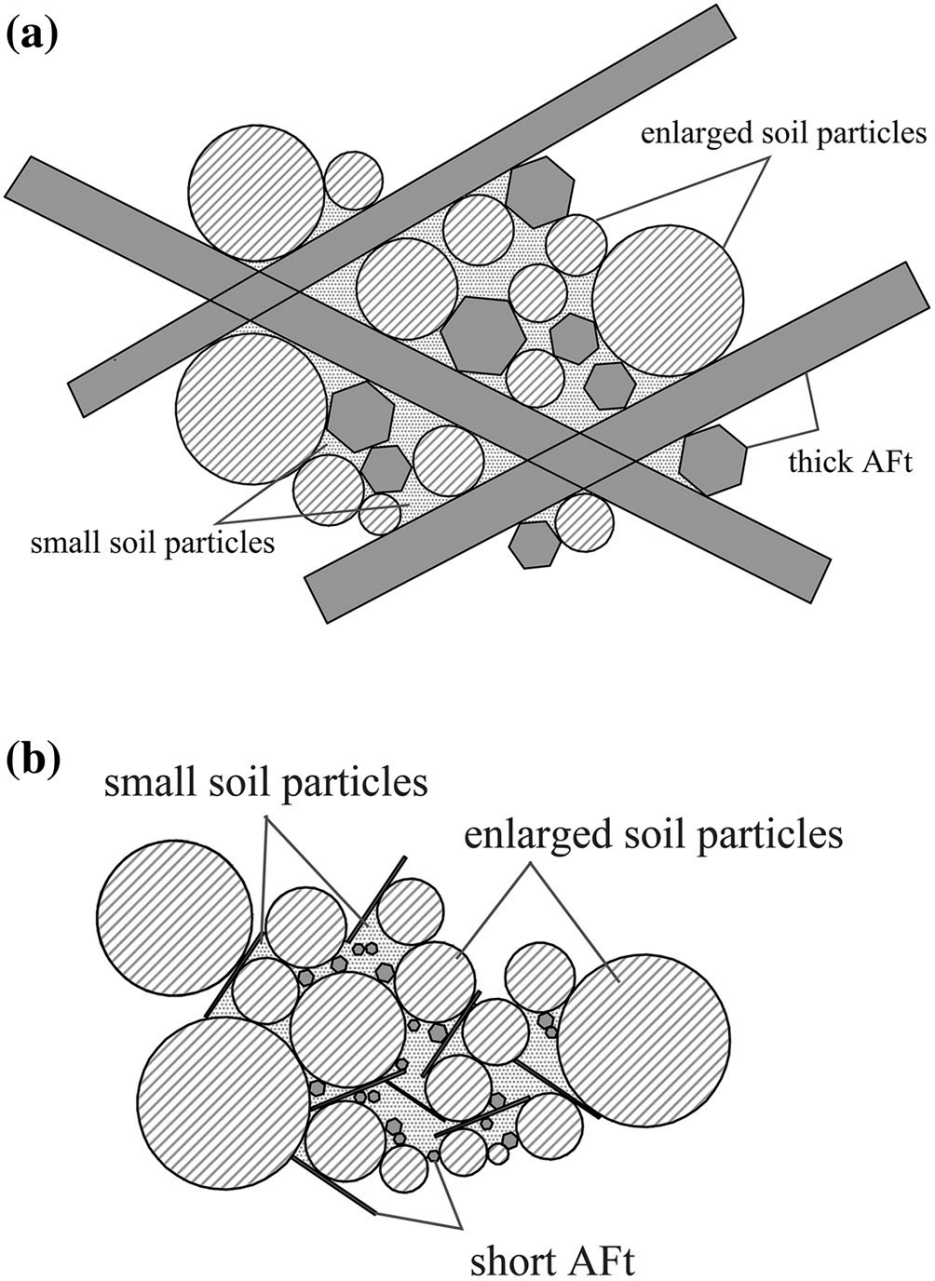
Microscopic model diagram: (**a**) SAC sample; (**b**) OPC sample.

Although both SAC and OPC improved greatly the mechanical properties of frozen soil, the former was significantly better than the latter. However, the SAC sample had a very close particle size distribution to that of the OPC sample (Fig. 13), when they were cured for 28 days. Their difference was mainly caused by the morphology of AFt. The AFt produced in SAC present pillared morphological characteristics. However, AFt produced in OPC present a short needlelike shape. Therefore, the difference in mechanical properties is likely to be because of the AFt. As shown in Fig. 14, the AFt acts as a supporting framework and results in a higher resistance to deformation and a higher strength. The thicker AFt crystals result in a higher strength. The above discussion demonstrates that AFt plays an important role in improving the mechanical properties of frozen soils.

For the SAC samples, the thawing deformation reduced and the strength increased with a monotonous tendency as the increase in dosage of cement. Although the maximum dosage studied in the tests is 15%, it seems to be the optimal content to improve the ice-rich frozen soils, in that the sample with 15% SAC after 28 curing days almost eliminated the thaw compression deformation and reached a strength meeting the rolling compaction requirement. Considering the slow growth on strength under the negative temperature condition, however, it may requires an appropriate increase in cement dosage for shortening construction period.

## Conclusions

In this study, three kinds of cements (SAC, OPC and MPC) were selected to improve frozen soil at negative temperature. The main objective was to study the change of strength and deformation behavior and explore the microscopic mechanism of improving process. The objective was achieved by carrying out mechanical tests and microstructural analyses on untreated and treated frozen soil with different dosages of cement and different curing ages. The conclusions of this research could be drawn as follows.

1. SAC is an effective stabilizer to improve frozen soil and 15% dosage is recommended. The OPC is not suitable to be used at negative temperature. MPC is an ineffective stabilizer at negative temperature.
2. The improvement of mechanical behaviors coms from two factors: the structural crystals and cementitious products. The two main factors contributed to the improvement of mechanical properties simultaneously. The thicker AFt crystals result in a higher strength and AFt plays an important role in improving the mechanical properties of frozen soils.
3. Although MPC produces a large of MKP crystals, the gel products did not agglomerate the soil particles well. The poor agglomeration on soil prevents MKP crystals from constructing a frame to sustain strength. Therefore, the strength of frozen soil had hardly improved.
4. The thaw compression deformation of frozen soil with water content 33% can be eliminate with 15% SAC. When the water content is less than 33%, the cement dosage can be appropriately reduced in terms of economy. Because temperature has a great influence on the cement hydration, the applicability of improved frozen soil in lower temperature construction environment or with higher water content will be further studied.
